# Load partitioning between the bcc-iron matrix and NiAl-type precipitates in a ferritic alloy on multiple length scales

**DOI:** 10.1038/srep23137

**Published:** 2016-03-16

**Authors:** Zhiqian Sun, Gian Song, Thomas A. Sisneros, Bjørn Clausen, Chao Pu, Lin Li, Yanfei Gao, Peter K. Liaw

**Affiliations:** 1 Department of Materials Science and Engineering, The University of Tennessee, Knoxville, Tennessee 37996, USA; 2 Materials Science and Technology Division, Los Alamos National Laboratory, Los Alamos, New Mexico 87545, USA

## Abstract

An understanding of load sharing among constituent phases aids in designing mechanical properties of multiphase materials. Here we investigate load partitioning between the body-centered-cubic iron matrix and NiAl-type precipitates in a ferritic alloy during uniaxial tensile tests at 364 and 506 °C on multiple length scales by *in situ* neutron diffraction and crystal plasticity finite element modeling. Our findings show that the macroscopic load-transfer efficiency is not as high as that predicted by the Eshelby model; moreover, it depends on the matrix strain-hardening behavior. We explain the grain-level anisotropic load-partitioning behavior by considering the plastic anisotropy of the matrix and elastic anisotropy of precipitates. We further demonstrate that the partitioned load on NiAl-type precipitates relaxes at 506 °C, most likely through thermally-activated dislocation rearrangement on the microscopic scale. The study contributes to further understanding of load-partitioning characteristics in multiphase materials.

Using precipitation strengthening^1^,^2^,^3^,^4^, coherent B2 NiAl-type precipitates have been employed to reinforce the body-centered-cubic (bcc) iron matrix^5^,^6^,^7^,^8^,^9^,^10^,^11^,^12^,^13^. Specifically, NiAl-type precipitates display the cubic-to-cubic crystallographic relation to the bcc-iron matrix. This type of ferritic alloys show promising creep resistance for high-temperature applications in fossil-energy power plants^5^,^8^,^10^. It has also been reported that NiAl-strengthened ferritic alloys suffer from room-temperature brittleness^6^,^14^,^15^,^16^,^17^, while Teng^18^ showed that the ductility can be improved by proper thermo-mechanical processing.

Strengthening mechanisms in materials containing reinforcing phases (such as NiAl-type precipitates in our case) can generally be classified into two types: (1) the resistance to dislocation movement by reinforcement/dislocation interactions through mechanisms, such as dislocation cutting, Orowan bowing, and dislocation climb^3^,^19^; and (2) the load-carrying capability of stiff inclusions due to elastic and plastic misfits among constituent phases^20^. The later mechanism plays an important role in mechanical properties of materials with a considerable volume fraction of strengthening phases. Load partitioning among constituent phases in engineering materials, such as carbon steels^21^,^22^,^23^,^24^,^25^, Al/SiC composites^26^,^27^, and nickel-based superalloys^28^,^29^, has attracted considerable attention. However, few studies have targeted the load-partitioning characteristics on multiple length scales (i.e., macroscopic, grain-level, and microscopic scales) and their relationship with mechanical properties.

In this study, we investigate the load-sharing behavior between the bcc-iron matrix and precipitates in a NiAl-strengthened ferritic alloy during uniaxial tension by methods including *in situ* neutron diffraction (ND) and slip-based crystal plasticity finite element (FE) model^22^,^30^. It is difficult to separate roles of individual phases in multiphase materials with only macroscopic stress-strain data. The ND combined with the constitutive modeling has been employed to elucidate the intergranular and interphase interactions in engineering materials^29^,^30^,^31^. We discuss factors of the strain-hardening behavior of the matrix, elastic and plastic anisotropy, and temperatures. By exploring load-partitioning features on multiple length scales and relationships with mechanical properties, our results further fundamental understanding of deformation mechanisms in multiphase materials, and provide insights into developing desirable multiphase engineering alloys or composites.

## Results

### Tensile stress vs. strain responses

The material used in the study is designated as FBB8 (Fe-6.66Al-10.29Ni-10.30Cr-3.57Mo-0.25Zr-0.005B, weight percent). See Materials preparation in Methods for details. Spherical NiAl-type precipitates are embedded in the matrix with a radius of ~54 nm and a volume fraction of ~15–16%^32^ after the 100-h aging treatment at 700 °C. The matrix/precipitate lattice mismatch was reported to be 0.02% at 700 °C^5^.

**Figure 1:**
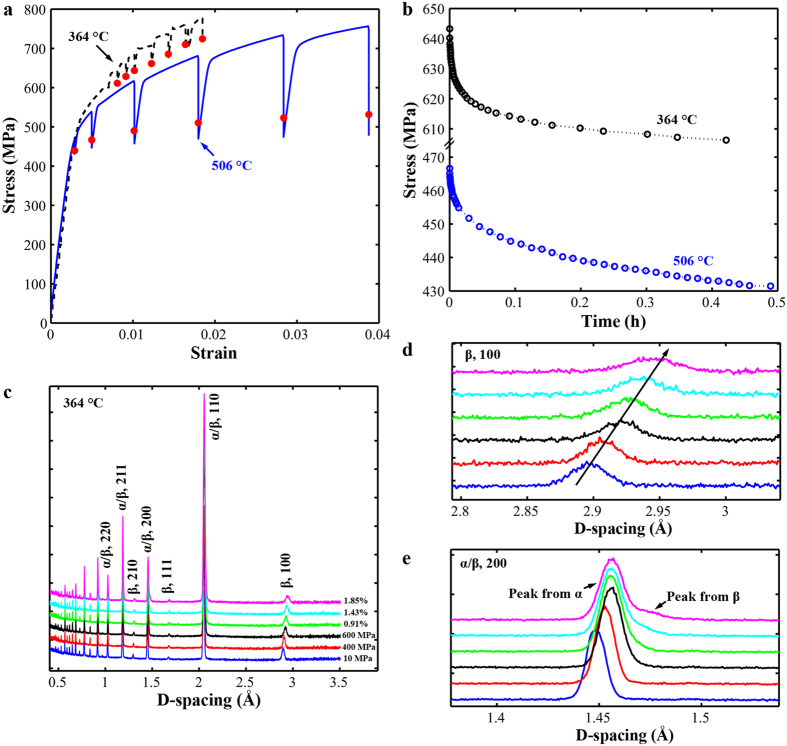
*In situ* neutron experiments. (**a**) Stress-strain curves recorded during *in-situ* tensile experiments. During neutron collecting periods, the load was kept constant in the elastic regime and the displacement was kept constant in the plastic regime. Average stresses over neutron-counting periods are marked as red dots. (**b**) First stress decay curves at both temperatures. (**c**) Representative axial diffraction patterns for the *in-situ* tensile test at 364 °C (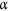: bcc-iron; *β*: NiAl-type precipitates). Enlarged evolutions of 100 superlattice reflection and 200 fundamental reflection are shown in (**d**) and (**e**), respectively.

Figure 1a displays the nominal stress-strain curves for uniaxial tensile tests at 364 and 506 °C. The step loading incorporated total 14 steps at 364 °C and 13 steps at 506 °C (see *In situ* neutron experiments in Methods). The specimens yielded at a stress between 500 to 600 MPa at 364 °C, and 450 to 500 MPa at 506 °C. The elastic moduli are estimated to be ~170 GPa at both temperatures by linear regression of data in the elastic regime. The applied load continues to increase in the plastic regime due to the strain hardening of the bcc-iron matrix and load carried by NiAl-type precipitates. Load slightly relaxes during neutron-counting periods with a fixed crosshead displacement at 364 °C. However, the decay is more pronounced at 506 °C than 364 °C as shown in Fig. 1a. Typical stress decay curves at both temperatures are demonstrated in Fig. 1b. Initially, stress drops rapidly, and then decay rates gradually slow down at both temperatures. The activation volume will be derived based on stress decay curves, as will be discussed later. The value of the average stress over neutron-counting periods (marked as red spots in Fig. 1a), defined as the integrated area below a stress decay curve divided by neutron-counting time, increases from 610 to 725 MPa as the test proceeds at 364 °C, while it saturates around ~530 MPa at macrostrain larger than 2.0% at 506 °C.

Representative axial diffraction spectra are exhibited in Fig. 1c for the test at 364 °C. Only results from lattice planes perpendicular to the loading axis are presented in this study. In Fig. 1c, we observe superlattice reflections from ordered B2 NiAl-type precipitates (e.g., 100, 111, and 210) as well as overlapped fundamental peaks (e.g., 110, 200, 211, and 220), which are common for the matrix and NiAl-type precipitates. Overall, peaks shift to large d-spacing values as applied stress increases (see the 100 reflection evolution in Fig. 1d). Since both phases have similar elastic stiffness^5^,^33^, fundamental reflections remain overlapped in the elastic regime. However, reflections gradually separate at the onset of macroscopic plasticity, indicating that load transfers to NiAl-type precipitates from the matrix. For an instance, as observed from the 200 reflection evolution in Fig. 1e, the overlapped 200 peak (~1.4472 Å prior to loading) gradually becomes separated in the plastic regimeat a strain of ~1.85% (~1.4560 Å for the bcc-iron matrix and 1.4728 Å for NiAl-type precipitates). The extent of peak separations strongly depends on crystallographic orientations relative to the loading axis.

### Lattice-strain evolutions

**Figure 2:**
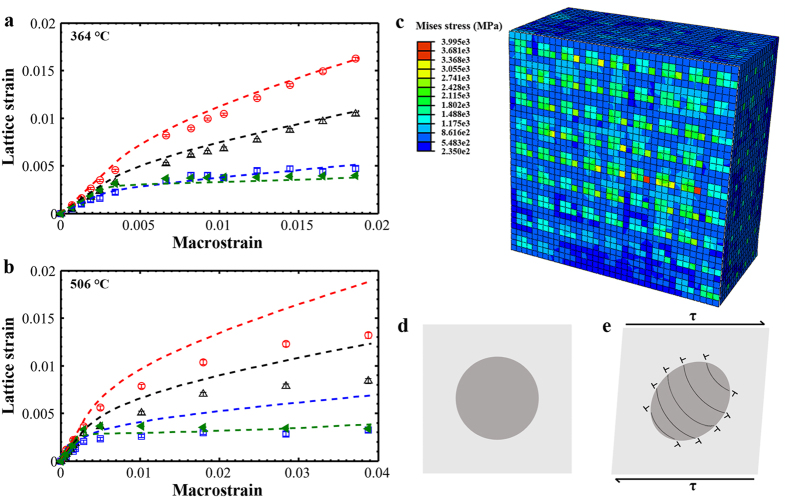
Lattice-strain evolutions. Axial lattice strains with macrostrain for the overall matrix and 100-, 111-, and 210-oriented precipitates relative to the loading axis at (**a**) 364 °C and (**b**) 506 °C (experimental results/FE predications: markers/lines; matrix: 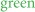; 100: 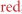; 111: 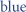; 210: black). (**c**) The crystal-plasticity FE modeling shows that NiAl-type precipitates carry a larger share of load in the plastic regime. (**d,e**) Illustration of dislocation structures around NiAl-type precipitates in the plastic regime.

Evolutions of axial lattice strains of the matrix and 100-, 111-, and 210-oriented NiAl-type precipitates relative to the loading axis (i.e., 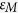, 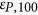, 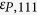, and 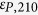) are shown as a function of macrostrain at both temperatures in Fig. 2a,b and Supplementary Fig. S2. At 364 °C (Fig. 2a and Supplementary Fig. S2a), 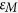 (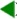) linearly increases with macrostrain in the elastic regime, results expected for materials obeying Hooke’s law. Linear regression of applied stresses vs. 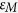 data in the elastic regime gives a diffraction elastic modulus of ~158 GPa. Upon macroscopic plasticity, 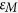 slightly increases to ~0.40%. Meanwhile, the lattice strains of NiAl-type precipitates (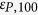: 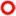, 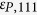: 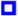, and 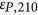: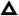) exhibit strong anisotropy in both elastic and plastic regimes (Fig. 2a). The values of diffraction elastic moduli of NiAl-type precipitates in 100, 111, and 210 directions are determined to be ~109, 228, and 154 GPa, respectively. In the plastic regime, 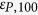 and 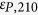 continue to increase as plastic flow continues. In contrast, 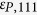 increases much more slowly to ~0.42%. The value of 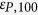 reaches as high as 1.62% at the macrostrain of 1.86%, approximately four times that of 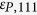.

At 506 °C (Fig. 2b and Supplementary Fig. S2b), NiAl-type precipitates show similar anisotropy in the elastic regime with diffraction elastic constants close to those at 364 °C. 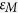 (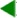) remains constant at ~0.35% after yield. Figure 2b also shows anisotropy of precipitate lattice strains in the plastic regime. The values of 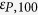 (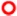) and 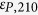 (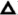) increase at the beginning and gradually become saturated (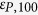: 1.3%; 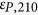: 0.84%). On the other hand, 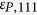 (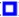) stays at ~0.29%.

We employed the crystal plasticity FE modeling to analyze the matrix/precipitate stress and strain distribution among grain families (see Crystal plasticity FE model in Methods). The term “grain families” refers to a set of grains which contribute to a specific hkl reflection in the axial direction. The values of the critical resolved shear stress (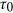) of the matrix were chosen to be 145 MPa at 364 °C and 130 MPa at 506 °C (Table 1), while precipitate elements were set to deform elastically. Load relaxation was not considered in the modeling. Figure 2c shows the Mises stress distribution in a cross section corresponding to the test at 364 °C with a macrostrain of ~1.86%. As can be seen in Fig. 2c, precipitate elements carry a larger share of load than surrounding matrix elements.

FE-modelled lattice strain evolutions with macrostrain are presented as lines at both temperatures in Fig. 2a,b. The FE predictions agree with experimental results at 364 °C (Fig. 2a), confirming that NiAl-type precipitates deform elastically at 364 °C. Upon plastic deformation, dislocation loops form around NiAl-type precipitates as illustrated in Fig. 2d,e 14, which accommodate the matrix/precipitate plastic misfit^20^. The stress on NiAl-type precipitates depends on the plastic misfit (Eq. S5) or equivalently dislocation arrangement around precipitates based on the Eshelby model^20^,^34^,^35^ (see Supplementary online). However, FE-modelled 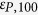, 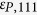, and 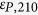 are larger than experimental values in the plastic regime at 506 °C (Fig. 2b), indicating load relaxation of NiAl-type precipitates during neutron-counting periods. We will explore possible deformation mechanisms in Discussion.

### Activation volume

**Figure 3:**
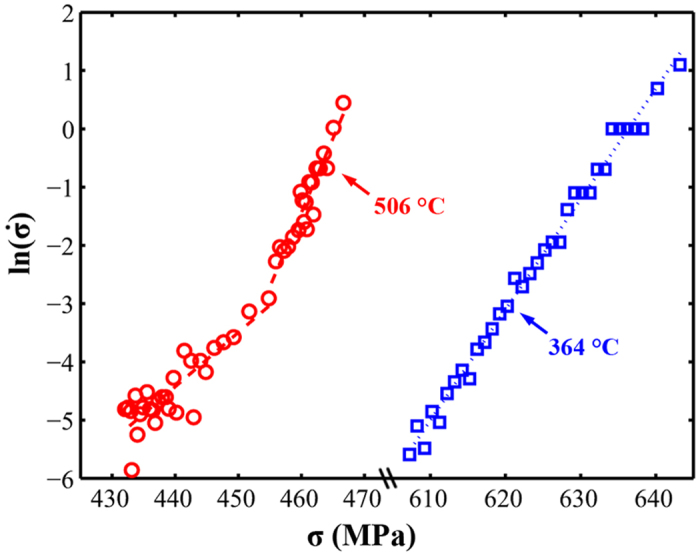
Activation volume. Ln(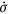) vs. *σ* obtained from the first stress decay curves (Fig. 1b) for both temperatures. The activation volume can be calculated by the slope between ln(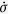) and *σ* based on Eq. (1).

The activation volume, 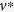, can be obtained from stress decay curves (Fig. 1b) through^36^,^37^


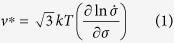


where k is the Boltzmann constant, T is the temperature, 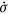 is the stress decaying rate, and σ is the applied stress. We derive the activation 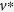 from the slope of 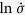 vs. 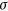 (Fig. 3) using Eq. 1. As shown in Fig. 3, the linear relationship between 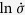 and 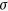 is well satisfied at 364 °C, giving an activation volume of ~183 b^3^, where b is the length of the Burgers vector (~2.508 Å at 364 °C and 2.514 Å at 506 °C, determined from the Rietveld refinement). However, two linear sections are observed at 506 °C, and the values of 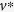 are estimated to be ~298 b^3^ from 467 to 455 MPa and 109 b^3^ from 455 to 433 MPa. For other stress decays at both temperatures, 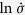 vs. 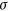 plots show similar trends and values of 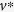 decrease with macrostrain probably due to the increasing dislocation density^38^. The observed 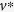 provides important information to distinguish deformation mechanisms in our case, as will be discussed later.

## Discussion

Assuming isotropic elasticity and spherical elastically-deforming precipitates, the mean stress, the difference between the average phase stress and applied stress, on NiAl-type precipitates in the loading direction, 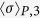, can be described based on the Eshelby model as


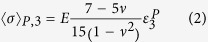


where E is the matrix elastic modulus, *ν* is the Poisson’s ratio, the subscript “3” indicates the loading direction, and 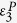 is the overall plastic strain with the superscript “P” indicating plastic deformation. According to Eq. 2, 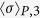 is expected to be linearly proportional to 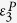. Taking E as 158 GPa and *ν* as 0.3, we found that 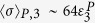 (GPa).

**Figure 4:**
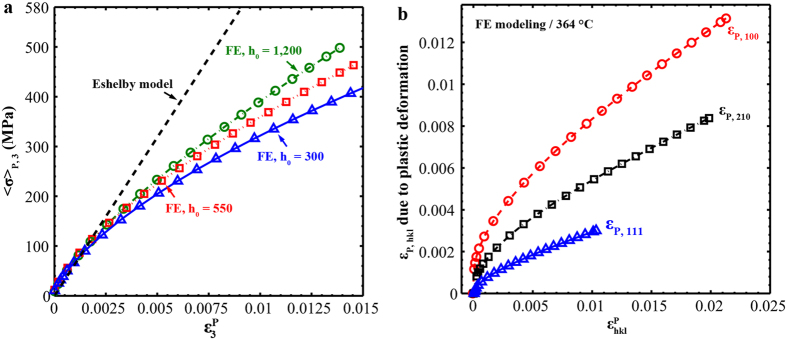
Load-partitioning characteristics on macroscopic and grain-level scales. **(a)** Mean stress on NiAl-type precipitates in the loading direction, 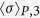, against the overall plastic strain, 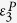, with the prediction by the Eshelby model. (**b)** Evolutions of *ε*_*P,hkl*_ due to plastic deformation with the plastic strains in 100, 111, and 210 grain families.

Using FE modeling, 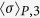 is plotted against 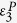 for three different h_0_ values (1,200, 550, and 300) together with the Eshelby prediction for comparison (Fig. 4a). h_0_ is related to the strain-hardening capability of the matrix (Eqs 6 and 7), and other parameters except h_0_ (Table 1) remain constant following the FE model at 364 °C. In Fig. 4a, 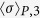 in all cases linearly increases against 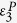 at the beginning (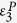 < ~0.1%) with a slope of ~60 GPa, as predicted by Eq. 2. However, 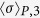 starts diverging downward from the trend line as plastic strain increases. Similarly, Oliver *et al.*^21^ found that stress on cementite gradually saturates after certain macrostrain in carbon steels. Moreover, the deviation from the Eshelby prediction depends on h_0_: given 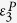, the value of 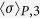 is larger for the case with stronger strain-hardening capability expressed as a larger h_0_. The matrix with more pronounced strain hardening produces larger elastic strain on NiAl-type precipitates in the plastic regime. Hence, we observe that the macroscopic load-transfer efficiency between the matrix and NiAl-type precipitates is not as high as that predicted by the Eshelby model and conclude that the difference is related to the matrix strain-hardening capability.

On the grain scale, the axial precipitate lattice-strain evolutions of 100, 111, and 210 grain families (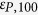, 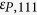, and 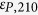) show strong anisotropy in the plastic regime (Fig. 2a,b). Young *et al.*^25^ also observed the anisotropic load carried by cementite in a carbon steel, even though reasons underneath the phenomenon remain obscure^24^. In Fig. 4b, 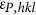 due to plastic deformation is plotted against the plastic strain in corresponding hkl grains families, 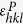. It demonstrates macroscopic inhomogeneous plastic deformation with plastic strains of ~2.1% for 100, 1.0% for 111, and 2.0% for 210 grain families. The overall plastic strain for the test at 364 °C is ~1.4%. We also note that 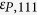 < 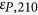 < 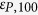 given a 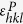, which results from the elastic anisotropy of NiAl-type precipitates. As stated, the diffraction elastic moduli of NiAl-type precipitates in 100, 111, and 210 directions are ~109, 228, and 154 GPa, respectively. Precipitates axially aligned in stiff directions (e.g., 111) are more resistant to elastic deformation enforced by the surrounding matrix than those axially aligned in compliant directions (e.g., 100)^20^. Therefore, we conclude that both the plastic anisotropy of the matrix and elastic anisotropy of NiAl-type precipitates contribute to the observed grain-scale anisotropic lattice-strain evolutions as shown in Fig. 2a,b.

The stress on the matrix and NiAl-type precipitates tends to decay during neutron-counting intervals as shown in Fig. 1a. The stress relaxation of the matrix is not captured well by ND at both temperatures, when compared with FE-modelled results (Fig. 2a,b), probably because the change of 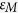 is beyond the instrumental resolution. The magnitude of the observed 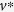 values is around several hundreds of b^3^ (109–298 b^3^) at both temperatures, as stated in Results, suggesting dislocation activities as dominant mechanisms. The diffusion-controlled creep and matrix/precipitate interfacial sliding can be ruled out, since the expected 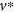 value is of the order of atomic volume (~1 b^3^) for either mechanisms^37^,^39^, which is much smaller than those we observed. Based on Vo and co-authors’ estimation^8^, the precipitate shearing stress of NiAl-type precipitates in FBB8 is ~1000 MPa due to the high anti-phase boundary energy (~0.5 Jm^−2^) in the (110) plane^40^. It is unlikely that dislocations in the bcc-iron matrix can cut through aged NiAl-type precipitates, which has been confirmed by detailed transmission electron microscopy (TEM) work^8^,^9^,^10^. The dislocation forest cutting or cross-slip in the matrix might control the stress relaxation at 364 °C and in the stress range of 467–455 MPa at 506 °C based on the activation volume^37^,^41^,^42^. Martin^43^ suggested that dislocation loops around inclusions are thermally instable due to high local stress produced on inclusions and surrounding matrix. At 506 °C, dislocations around NiAl-type precipitates are likely to rearrange themselves through thermally-activated climb and slip to reduce the matrix/precipitates plastic misfit, leading to the load relaxation of NiAl-type precipitates, as observed in Fig. 3b. Microstructural characterization by TEM would reveal details of dislocation activities.

In regard to the creep behavior of FBB8, Huang *et al.*^5^ performed *in situ* creep study of FBB8 at 700 °C/107 MPa under ND and they did not observed the load transferring from the matrix to NiAl-type precipitates. By combining their results, we suggest that the precipitate load-carrying capability in FBB8 is limited under expected service conditions in fossil-energy power plants as long as dislocation activities (e.g., dislocation climb) control the deformation. First of all, the load-transfer efficiency to precipitates is low due to weak strain hardening of the bcc-iron matrix at high temperatures (≥650 °C^44^) (Fig. 4a). Next, the stress on NiAl-type precipitates tends to relax through dislocation rearrangement. In this scenario, the presence of reinforcements may not be effective to decrease creep rate as reinforcements cannot carry the larger share of load under certain conditions^27^. Krug and Dunand^45^ concluded that the threshold stress for the dislocation-climb-controlled creep is proportional to the matrix/precipitate lattice mismatch. We might be able to control the matrix/precipitate mismatch by adjusting compositions of NiAl-strengthened ferritic alloys^46^ in order to increase the matrix/precipitate interaction, and consequently the load carried by NiAl-type precipitates. Meanwhile, we need to keep the matrix/precipitate interface coherent considering the precipitate stability^46^,^47^,^48^,^49^ for possible long-term applications at high temperatures.

We have investigated the load partitioning between the bcc-iron matrix and NiAl-type precipitates in FBB8 during the uniaxial tensile tests at 364 and 506 °C. Load transfers from the plastically-deforming matrix to precipitates at both temperatures. The macroscopic load-transfer efficiency depends on the matrix strain-hardening behavior, and is not as high as that predicted by the Eshelby model. On the grain-level scale, the anisotropic load-partitioning behavior among grain families is attributed to the plastic anisotropy of the matrix and elastic anisotropy of NiAl-type precipitates. Microscopically, part of load on NiAl-type precipitates relaxes during neutron-counting intervals at 506 °C probably through local dislocation arrangement based on the activation volume. Our results profile and advance our understanding of the load-partitioning characteristics on macroscopic, grain-level, and microscopic scales, which help guide design and development of desirable alloys or composites with multiple phases.

## Methods

### Materials preparation

A NiAl-strengthened ferritic alloy, designated as FBB8 (Fe-6.66Al-10.29Ni-10.30Cr-3.57Mo-0.25Zr-0.005B, weight percent), was investigated in this study. Ingots (~12.7 × 25.4 × 1.9 cm) were prepared by the Sophisticated Alloys, Inc., using vacuum induction melting. Hot isostatic pressing (1,200 °C/103 MPa/4 h) minimized casting porosity. The ingot composition was determined using a direct current plasma-atomic emission spectrometry (DCP-AES) by Sherry Laboratories. Specimens encapsulated into evacuated quartz tubes were solution treated at 1,200 °C for 1 hour, followed by air cooling, and then aged for 100 h at 700 °C. The average grain size was ~100 μm.

### *In situ* neutron experiments

Two threaded tensile specimens of 41.28 mm in gauge length and 6.35 mm in diameter were *in situ* loaded in a vacuum at the Spectrometer for the Materials Research at Temperature and stress (SMARTS) of the Los Alamos Neutron Science Center (LANSCE), the Los Alamos National Laboratory. Detailed descriptions of the experimental setup at SMARTS can be found elsewhere^50^,^51^,^52^. Sample temperatures, measured by thermocouples attached to specimens, were 364 and 506 °C, respectively^7^,^14^,^16^,^17^. Step loading was used (Supplementary Fig. S1) with a preload of ~10 MPa: there were total 14 steps (constant load: 10, 100, 200, 300, 400, 500, and 600 MPa; constant displacement: 0.81%, 0.91%, 1.02%, 1.23%, 1.43%, 1.64%, and 1.85%) at 364 °C and 13 steps at 506 °C (constant load: 10, 100, 150, 200, 250, 300, and 350 MPa; constant displacement: 0.29%, 0.50%, 1.02%, 1.80%, 2.84%, and 3.88%). Time-of-flight diffraction spectra from lattice planes normal (axial) and parallel (transverse) to the loading axis were acquired by two sets of detectors with a count time of half an hour. The diffraction spectra represented an average of information in counting periods. Macrostrain was monitored by a high-temperature extensometer. Due to limited beam time, only one test was performed for each temperature, and thus, there may be certain uncertainty in experiments results.

The lattice constant, a, was obtained by the Rietveld refinement of complete spectra using the General Structure Analysis System (GSAS) package^53^, while the single-peak-fitting procedure in GSAS was employed to determine the d-spacing of hkl lattice planes, d_hkl_. Note that only axial spectra from lattice planes normal to the loading axis were analyzed and presented in this study. Two kinds of lattice strains were calculated: (1) axial elastic phase strain of the bcc-iron matrix (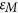), defined as


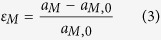


where 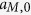 is the reference matrix lattice constant prior to loading; and (2) axial hkl lattice strain of NiAl-type precipitates, defined as


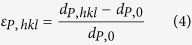


where 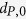 is the reference d-spacing of hkl lattice planes in NiAl-type precipitates prior to loading. The subscript, “M”, stands for the matrix, and “P” for NiAl-type precipitates.

### Crystal plasticity FE model

We used the commercial software Abaqus 6.11 to implement crystal plasticity FE modeling. The Abaqus user-defined material (UMAT) subroutine developed by Huang^54^ was modified for the lattice-strain calculation^30^. In the model, the power law is used to describe the strain-rate dependence


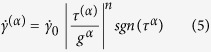


where *α* represents the αth slip system, 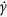 is the shear strain rate, 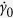 is a characteristic strain rate, *τ* is the resolved shear stress, g is the current strength of the slip system, and n is the stress exponent.

The strain-hardening behavior is expressed through the incremental relation


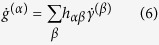


where 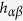 is the slip-hardening moduli, and the sum includes all activated slip systems. For the self-hardening moduli, 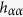, it follows the form proposed by Peirce, Asaro, and Needleman^55^, and Asaro^56^,^57^


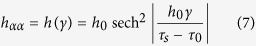


where 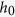 is the initial hardening modulus, 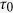 is the yield stress, 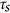 is the saturated stress, and *γ* is the Taylor cumulative shear strain on all slip systems, while the latent hardening moduli are given by


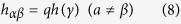


where q is the latent hardening constant.

A total of 729 (9 × 9 × 9) randomly-oriented cubic grains were used in the model (Supplementary Fig. S3a). Each grain consisted of 64 elements, and ten of them (eight at the center plus two on the surface) were assigned to be NiAl-type precipitates. Since elastic properties of the matrix and precipitates at both temperatures are similar, the same elastic constants (C_11_, C_12_, and C_44_) were used in all models. The elastic constants were estimated, following Dever’s work^58^ and further adjusted by comparing with experimental diffraction elastic constants. Precipitate elements were set to deform elastically, and therefore, only elastic constants were meaningful to these elements. Parameters, listed in Table 1, were chosen so that the modelled tensile stress-strain curves follow the experimental results (the original applied stress vs. macrostrain), as shown in Supplementary Fig. S3b.

## Additional Information

**How to cite this article**: Sun, Z. *et al.* Load partitioning between the bcc-iron matrix and NiAl-type precipitates in a ferritic alloy on multiple length scales. *Sci. Rep.* **6**, 23137; doi: 10.1038/srep23137 (2016).

## Supplementary Material

Supplementary Information

## Figures and Tables

**Table 1:** Parameters used in the crystal plasticity FE modeling.

	C_11_ (GPa)	C_12_ (GPa)	C_44_ (GPa)	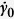	n	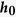	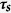 (MPa)	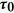 (MPa)	q
364 °C	183.2	115.5	97.2	0.0001	10	500	350	145	1
506 °C	183.2	115.5	97.2	0.0001	10	100	250	130	1

Note only elastic constants (C_11_, C_12_, and C_44_) are meaningful for precipitate elements, since they were set to deform elastically.
