# Supplementary material for: Load partitioning between the bcc-iron matrix and NiAl-type precipitates in a ferritic alloy on multiple length scales
Source: Sci Rep. 2016 Mar 16;6:23137. doi: 10.1038/srep23137 (PMC4793255; doi:10.1038/srep23137)
Supplement: Supplementary Information [file srep23137-s1.pdf]

Supplementary information for

Load partitioning between the bcc-iron matrix and NiAl-type precipitates in a ferritic alloy on multiple length scales

Zhiqian Sun[1], Gian Song[1], Thomas A. Sisneros[2], Bjørn Clausen[2], Chao Pu[1], Lin Li[1],

Yanfei Gao[1], and Peter K. Liaw[1*]

1. Department of Materials Science and Engineering, The University of Tennessee, Knoxville, Tennessee 37996, USA

2. Materials Science and Technology Division, Los Alamos National Laboratory, Los Alamos, New Mexico 87545, USA

* Correspondence and questions regarding the manuscript should be addressed to Dr. Peter K. Liaw at pliaw@utk.edu.

Eshelby model

Assuming the isotropic elasticity and randomly-distributed precipitates, the macroscopic load transfer to precipitates can be estimated by the Eshelby model. Based on the Eshelby model[1-3], the plastic misfit between the matrix and NiAl-type precipitates, ε^{T^*}, can be expressed as

$$\varepsilon^{T^*} = -\varepsilon^{P}_{M,3}\left(-\frac{1}{2}, -\frac{1}{2}, 1, 0, 0, 0\right)^{T} \quad \text{(S1)}$$

where $\varepsilon^{P}_{M,3}$ is the plastic strain of the matrix in the loading direction ("3" axis). The subscript, "M", stands for the matrix, and the subscript, "P", for NiAl-type precipitates. The superscript, "P", indicates plastic deformation. $\varepsilon^{P}_{M,3}$ can be associated with the overall plastic strain, ε^{P}_{3}, by

$$\varepsilon^{P}_{3} \sim (1-f)\,\varepsilon^{P}_{M,3} \quad \text{(S2)}$$

The equivalent transformation strain, ε^{T}, is defined as

$$\varepsilon^{T} = -\{(C_M - C_P)[S - f(S - I)] - C_M\}^{-1} C_P \varepsilon^{T^*} \quad \text{(S3)}$$

where C_M and C_P are the stiffness tensors of the matrix and precipitates, respectively, I is the identity matrix, and S is the Eshelby tensor and is given by Clyne and Withers[1] for spherical inclusions. By assuming that the matrix and precipitates have the same stiffness tensor, Eq. (S3) is simplified as follows

$$\varepsilon^{T} = \varepsilon^{T^*} \quad \text{(S4)}$$

The mean stress on precipitates can be expressed, using ε^{T}, by

$$\langle\sigma\rangle_P = (1-f)C_M(S - I)\varepsilon^{T} \quad \text{(S5)}$$

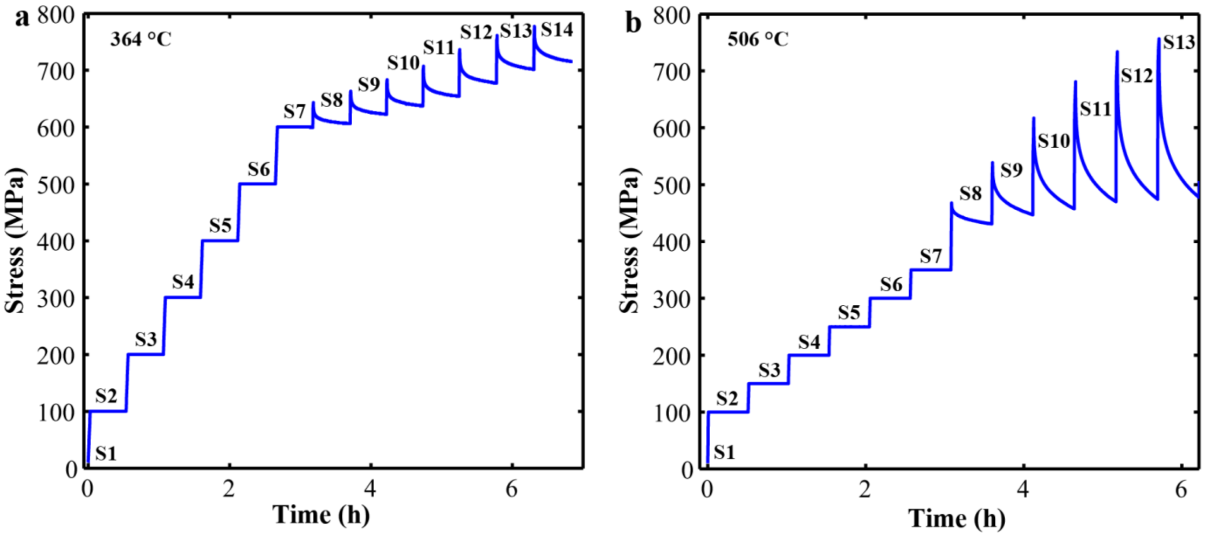

Figure S1. Step-loading tensile tests. Step loadings during in-situ neutron experiments at (**a**) 364 ℃ and (**b**) 506 ℃. Steps [S1 – S7 in **(a)** and **(b)**] are stress-controlled; while steps [S8 – S14 in **(a)** and S8 – S13 in **(b)**] are displacement-controlled.

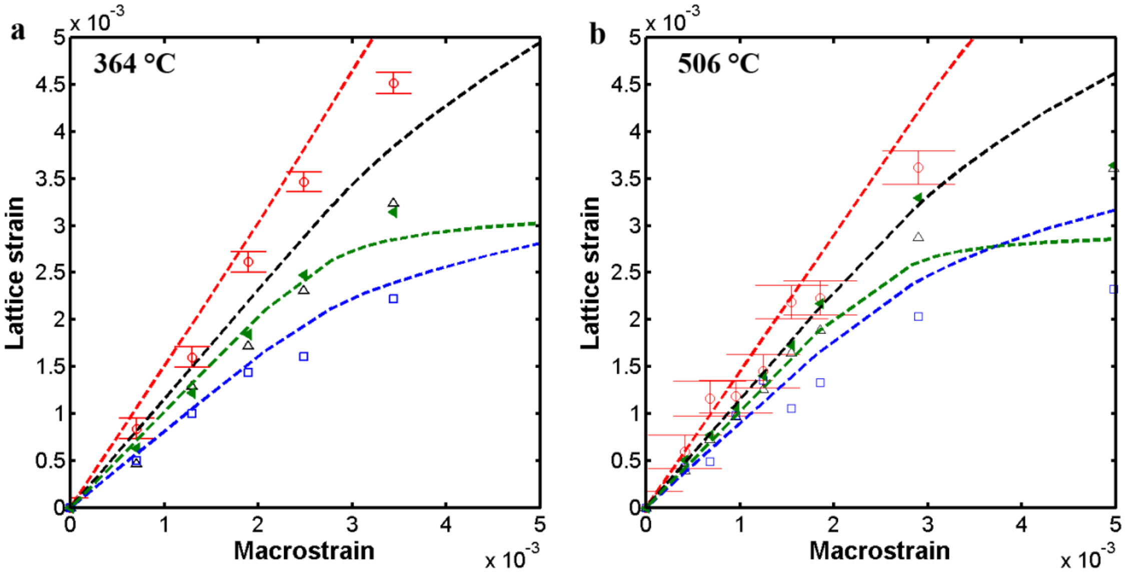

Figure S2. Lattice-strain evolutions in the elastic regime. Axial lattice strains with macrostrain for the overall matrix and 100-, 111-, and 210-oriented precipitates relative to the loading axis at (**a**) 364 ℃ and (**b**) 506 ℃ (experimental results/FE predications: markers/lines; matrix: green; 100: red; 111: blue; 210: black).

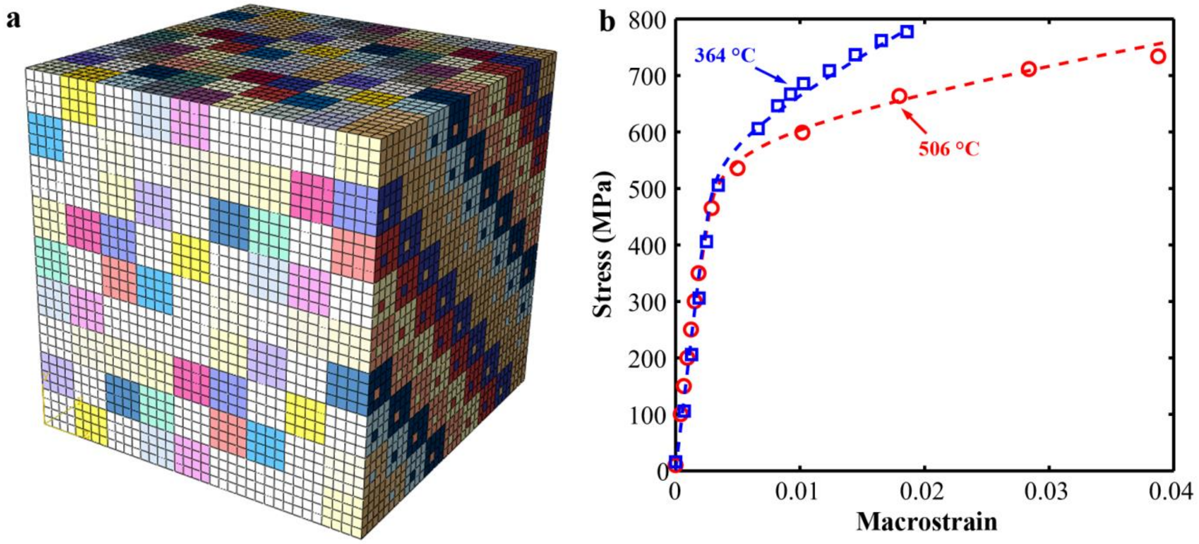

Figure S3. Crystal-plasticity FE model. (**a**) Whole element sets in the crystal-plasticity FE model. Cubic grains are distinguished by different colors. Parameters were chosen so that modelled tensile curves (lines) followed experimental results (unrelaxed stress-strain for each step) (markers) in (**b**).

References

1 Clyne, T. W. & Withers, P. J. *An Introduction to Metal Matrix Composites*. 130-141 (Cambridge University Press, 1993).

2 Eshelby, J. D. The determination of the elastic field of an ellipsoidal inclusion, and related problems. *Proc. Roy. Soc. (London) A* **241**, 376-396 (1957).

3 Mori, T. & Tanaka, K. Average stress in matrix and average elastic energy of materials with misfitting inclusions. *Acta Metall.* **21**, 571-574 (1973).
